# The chemical tool-kit for molecular imaging with radionuclides in the age of targeted and immune therapy

**DOI:** 10.1186/s40644-021-00385-8

**Published:** 2021-01-30

**Authors:** Timothy H. Witney, Philip J. Blower

**Affiliations:** School of Biomedical Engineering and Imaging Sciences, King’s College London, 4th Floor Lambeth Wing, St Thomas’ Hospital, London, SE1 7EH England

**Keywords:** PET, SPECT, Molecular imaging, Radiochemistry, Radiopharmaceuticals, Total body PET, Radionuclides

## Abstract

Nuclear medicine has evolved over the last half-century from a functional imaging modality using a handful of radiopharmaceuticals, many of unknown structure and mechanism of action, into a modern speciality that can properly be described as molecular imaging, with a very large number of specific radioactive probes of known structure that image specific molecular processes. The advances of cancer treatment in recent decades towards targeted and immune therapies, combined with recognition of heterogeneity of cancer cell phenotype among patients, within patients and even within tumours, has created a growing need for personalised molecular imaging to support treatment decision. This article describes the evolution of the present vast range of radioactive probes – radiopharmaceuticals – leveraging a wide variety of chemical disciplines, over the last half century. These radiochemical innovations have been inspired by the need to support personalised medicine and also by the parallel development in development of new radionuclide imaging technologies – from gamma scintigraphy, through single photon emission tomography (SPECT), through the rise of clinical positron emission tomography (PET) and PET-CT, and perhaps in the future, by the advent of total body PET. Thus, in the interdisciplinary world of nuclear medicine and molecular imaging, as quickly as radiochemistry solutions are developed to meet new needs in cancer imaging, new challenges emerge as developments in one contributing technology drive innovations in the others.

## Introduction

**Fig. 1:**
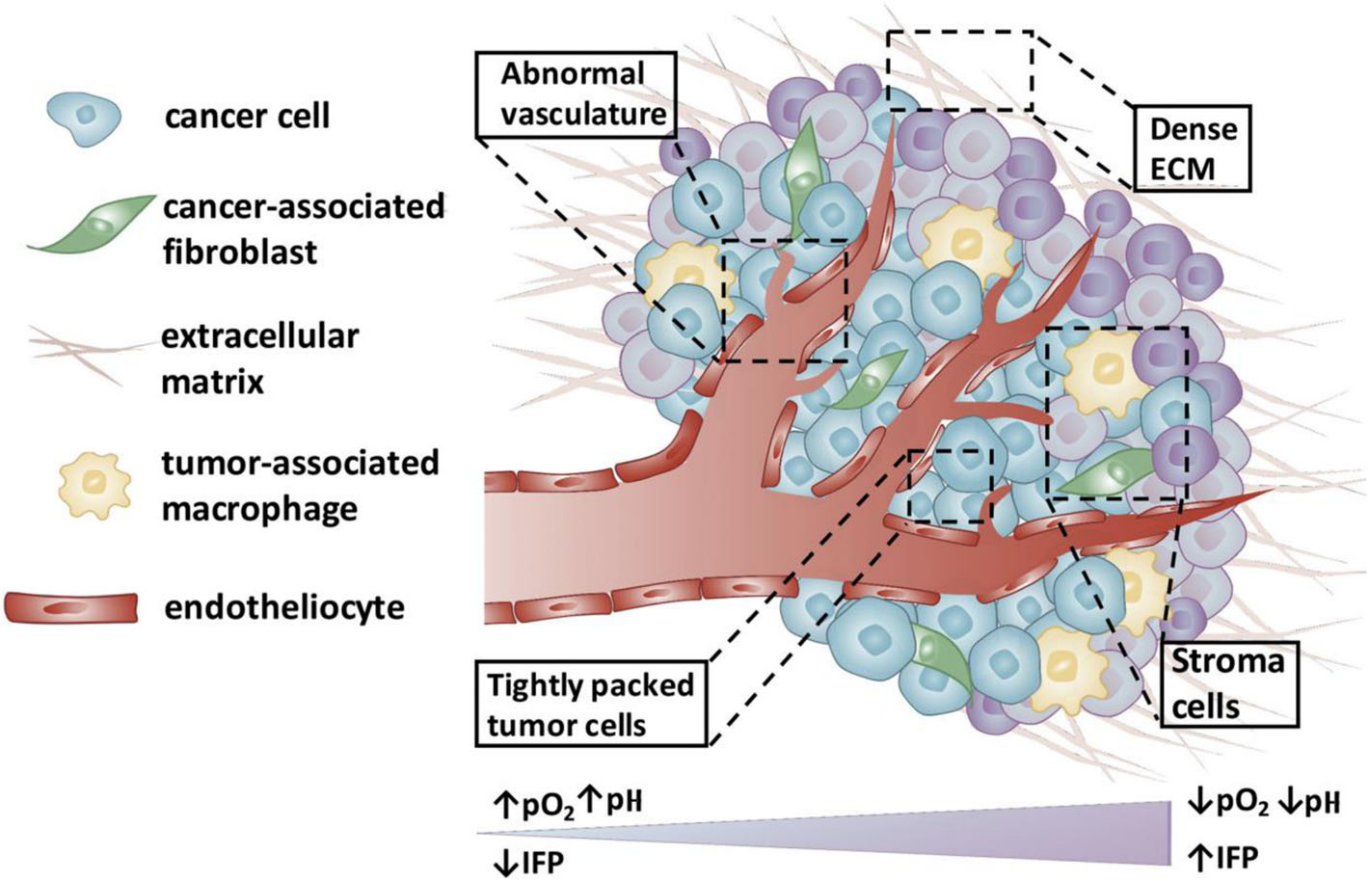
A simplified illustration of the tumour microenvironment. Modified and reprinted from Overchuk et al. [2]

Advances in gene sequencing and single cell transcriptomics have revolutionised our understanding of clonal heterogeneity and cancer evolution [1]. These next-generation sequencing techniques, applied to tumour biopsy samples, clearly illustrate the complexity and interconnectivity of the tumour microenvironment; tumour cells with distinct molecular signatures dynamically interact with cells from the innate and adaptive immune system, tumour-associated fibroblasts and their surrounding extracellular matrix (Fig. 1). These interactions evolve as disease progresses and generates genetic mutations, resulting in extensive intra- and inter-tumoural heterogeneity. In the modern age of ‘precision medicine,’ it therefore seems antiquated that tumour classification (and resulting treatment decisions), often based on a single biopsy which samples a tiny fraction of the disease at a fixed time point, determines treatment decisions. Rather, what is required is continual monitoring of the disease, across regions within tumours, among different lesions across the whole body, and over time. Despite the power of sequencing and transcriptomics, biopsy sampling, being invasive and intrinsically limited to small samples, cannot provide this.

On the other hand, molecular imaging techniques, such as positron emission tomography (PET) and single photon emission computed tomography (SPECT), present the opportunity to spatiotemporally monitor, in the living patient, the expression and activity of key biomarkers associated with the cancer phenotype. Direct conjugation of positron or gamma-emitting radionuclides to small molecule substrates, inhibitors, and larger biomolecules such as peptides, antibodies, and even whole cells, has enabled visualisation, repeatedly and across the whole body, of a wide variety of biological processes in living subjects. Moreover, the substitution of an imaging radionuclide for a therapeutic radioisotope (through alpha- or beta-particle emission) has been effectively used for molecular-targeted cancer treatment. Nuclear medicine imaging techniques, however, have been traditionally difficult to multiplex and hence cannot probe large numbers of different processes/genes, restricting the longitudinal monitoring of disease progression often to a single biomarker. In this review, we explore how recent developments in radiochemistry, alongside scanner development, have evolved from early functional imaging radiopharmaceuticals to provide the current tool-kit to image a wide range of molecular processes and gene expressions - “molecular imaging” – and how further development may overcome this limitation to herald a new era of molecular *systems* imaging.

### Imaging the immune microenvironment

Immunotherapy has led to durable, and in some cases, curative responses in patients with non-solid tumours [3]. The primary goal of these biological therapies is to induce sustained anti-tumour immunity through adoptive transfer of T-cells or enhancement of existing anti-tumour immune responses through targeting immune checkpoint pathways. Molecular imaging of these processes has provided new outlets for probe development and has already been shown to be superior to gold standard biomarker assay response prediction [4]. However, a reductive, single-biomarker imaging approach is unlikely to provide a comprehensive picture of tumour-immune cell interactions and tumour heterogeneity. Key questions explored through molecular imaging of immunotherapy relate to: 1. the activation status of key immune-mediators, such as T-cells; 2. metabolic signatures of tumour-specific immune cells; 3. expression of cell surface markers of immune populations and regulatory pathways; 4. the location and tumour-targeting of adoptively transferred cells; 5. pharmacokinetics of immunotherapies; and 6. accurate monitoring of tumour response to treatment [5]. Answering these questions through molecular imaging requires a wide array of probes, each presenting different radiochemical challenges. These range from small molecules/metabolites (where structural modification must be minimised), peptides (which can tolerate bioconjugation with small prosthetic groups), proteins and nanoparticles (which can often tolerate multiple conjugation/modification), cells (which require the gentlest labelling procedures) and drug delivery systems (e.g. liposomes).

### The sodium-iodide symporter – a paradigm for the evolution of molecular imaging

Eighty years on from the first administration of radioiodine to a human subject, [6] the human sodium-iodide symporter has become a paradigm for the evolution of nuclear medicine and radiopharmaceuticals; from crude tools to assess physiological function, towards precision measurement and quantitative imaging of molecular processes and their role in disease. Imaging and treatment of thyroid cancer and other thyroid disease with radioiodine is the archetypal theranostic combination of treatment with companion diagnostic, beginning with use of iodine-131, in the form of iodide ions, as both treatment (exploiting its beta-emission) and diagnostic (by imaging its gamma emissions). Later, new radioisotopes of iodine emerged, including iodine-123 which provided better quality planar scintigraphic (and later still, tomographic - SPECT) images and more favourable dosimetry. More recently, the benefits of PET – superior image quality, sensitivity and resolution - have been realised as the positron emitting radioisotope iodine-124 has become commercially available [7]. For the first half-century of clinical use of radioiodines, they could properly be described as “functional” imaging agents, based on knowledge that thyroid and thyroid cancer (and other specific tissues) were able to accumulate iodide, allowing measurement of function via the amount of radioactivity accumulated. With the identification and cloning, as recently as 1996, [8] of the transporter responsible for this accumulation – the sodium-iodide transporter, NIS (hNIS in its human form), it became meaningful to apply the newly-emerging term *molecular imaging* to the use of radioiodines in this context.

**Fig. 2:**
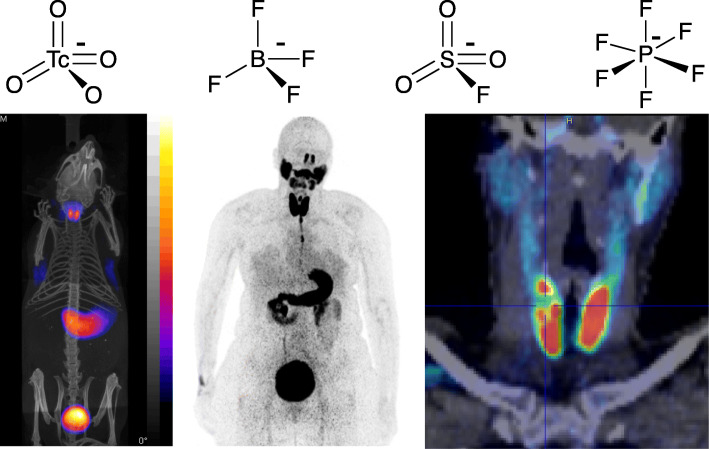
Imaging the sodium-iodide symporter. Top: Synthetic radiolabelled substrates of the sodium-iodide symporter hNIS used for radionuclide imaging. Left to right: [^99m^Tc]pertechnetate; [^18^F]tetrafluoroborate; [^18^F]fluorosulfate; [^18^F]hexafluorophosphate. Bottom: Images show [^18^F]tetrafluoroborate PET images of healthy mouse (left), human with unresected thyroid cancer (centre) and, in the same subject, enlarged coronal section of thyroid region showing “cold” region

However, the accumulation of iodine in thyroid tissue is not solely dependent on the activity of hNIS; hNIS mediates the initial entry of iodide ions into the thyrocyte, but its retention in and efflux from thyroid tissue is mediated by other processes including transport into the thyroid “colloid,” oxidation by peroxidase, incorporation into thyroid hormones, and later release of these hormones [9]. Thus, radioiodide imaging is not a simple mapping of hNIS activity. To perform true molecular imaging of hNIS, tracers are required that are substrates of hNIS but not of the subsequent iodine-processing cellular machinery. The prototypical example of this is technetium-99 m pertechnetate, a small anion resembling iodide sterically and electrostatically (Fig. 2) which has been used for thyroid imaging using scintigraphy and SPECT for many years. Conveniently, [^99m^Tc]pertechnetate is the chemical form of technetium-99 m eluted from the generator, hence no further chemistry development was required to apply this in the clinic.

Until recently, the main use of radioactive hNIS substrates for imaging has been to detect thyroid cancer and inform decisions about its treatment with radioiodine. A new role of molecular imaging as a tool to support modern targeted and immunotherapy emerges from use of hNIS as a reporter gene to track cells administered as part of cell-based immunotherapy regimes, such as chimeric antigen receptor (CAR) T-cell therapies [10]. By including hNIS expression alongside the genetic modifications that endow these cells with targeted therapeutic capability, they can be tracked by imaging with radioactive hNIS substrates, to determine their fate, location, survival and proliferation in the treated patient and in preclinical animal models. Radioiodines can be used for this purpose (iodine-123 for SPECT, iodine-124 for PET) but the sequestration of radioiodine in thyroid tissue due to the combination of hNIS expression and thyroid hormone synthesis reduces availability of radioiodide for targeting the hNIS-expressing therapeutic cells [9]. [^99m^Tc]pertechnetate is therefore more suitable for this reporter gene application, [11] and this has prompted chemists to develop new tracers that, like pertechnetate, are non-metabolisable hNIS substrates to extend this from SPECT to PET to achieve the improved sensitivity and quantification required in this new application. This has recently been achieved with a range of fluorine-18-labelled small anions [^18^F]tetrafluoroborate, [9, 12, 13] [^18^F]fluorosulfate [14] and [^18^F]hexafluorophosphate [15] (Fig. 2). Of these, [^18^F]tetrafluoroborate is in the vanguard of clinical and preclinical use and has been evaluated in healthy human volunteers, [16] thyroid cancer patients, [12] and preclinical models of cell tracking [9] and gene therapy [17]. These novel tracers extend true hNIS molecular imaging into the remit of PET. They also show that fluorine-18, the most important PET radionuclide and the radiolabel incorporated into hundreds of PET radiotracers via carbon-fluorine bonds, can be incorporated into radiopharmaceuticals by binding to elements other than carbon. This theme will recur later in the article. Reporter gene imaging with these hNIS substrates has shown promise in preclinical models of gene therapy [17] and cell tracking [9] but has yet to be translated to the clinic. The hNIS imaging story thus spans the entire history of nuclear medicine; it illustrates the historic transitions from functional to molecular imaging and from scintigraphy to PET and the way in which new tracer development supports and partners the development of advanced medicines.

### FDG and fluorine-18

**Fig. 3:**
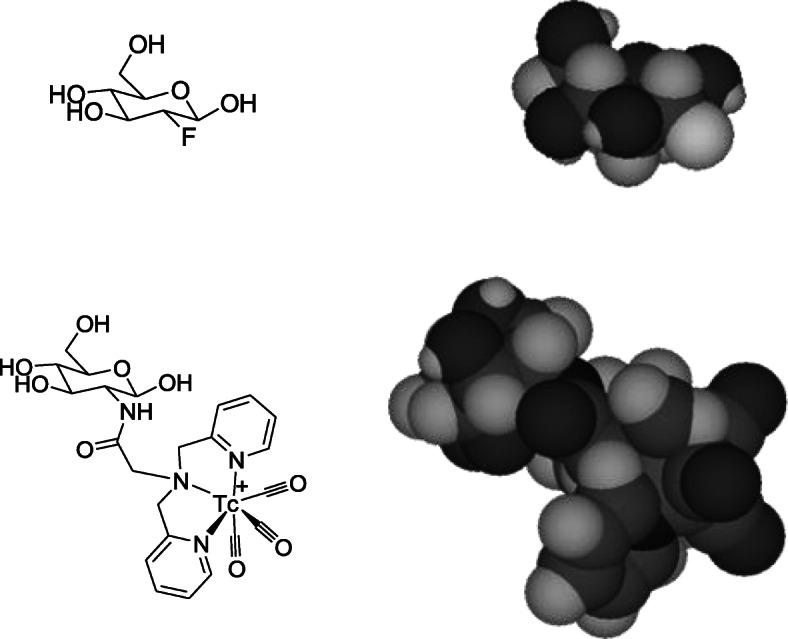
Radiotracers based on glucose structure (line diagrams and space-filling representations) comparing labelling with ^18^F (via C-F bond, in the form of fluorodeoxyglucose, FDG) (top); and with ^99m^Tc (via bifunctional chelator as exemplar radiometal conjugate). The large size of the ^99m^Tc complex makes it an unlikely substrate for GLUT-1 or hexokinase

The foregoing discussion highlights the importance, which cannot be overestimated, of fluorine-18. Indeed, the growth of PET as a ubiquitous clinical imaging modality over the last three decades can be clearly attributed to the invention of a single pivotal radiopharmaceutical, [^18^F]-2-fluoro-2-deoxyglucose (FDG) [18]. Its wide clinical utility and application in huge numbers of cancer patients has justified vast expenditure to install cyclotrons, radiochemistry facilities and PET scanners in hospitals worldwide. Fluorine-18 is critical to this development because it *uniquely* can be incorporated into a glucose-like molecule that can be used to image uptake of glucose via GLUT-1 (glucose transporter-1) and its trapping by hexokinase-catalysed phosphorylation [19]. Other radioelements capable of forming covalent bonds in small organic molecules are unsuitable. Radioiodines, for example, can be incorporated by C-I bond formation but not with the requisite resistance to deiodination. Numerous attempts to develop glucose conjugates with chelates of technetium-99 m and other radiometals have stumbled at validation as substrates of GLUT-1 or hexokinase (Fig. 3) [20]. Since only fluorine-18 can be used in this way, the PET infrastructure became indispensable despite its expense. Once this infrastructure became established, the scene was set for new ^18^F-labelled radiopharmaceuticals to be developed offering greater selectivity for cancer-related targets other than the ubiquitous glucose metabolism. Moreover, the new infrastructure could be used to produce other radionuclides (such as ^11^C, ^64^Cu, ^13^N, ^15^O, ^64^Cu), and scanners could be used with non-locally produced, longer half-life PET radionuclides (iodine-124, zirconium-89) and generator-produced radionuclides such as rubidium-82 and gallium-68, to exploit the superior imaging capability offered by PET. We will return later to some of these radionuclides.

**Fig. 4:**
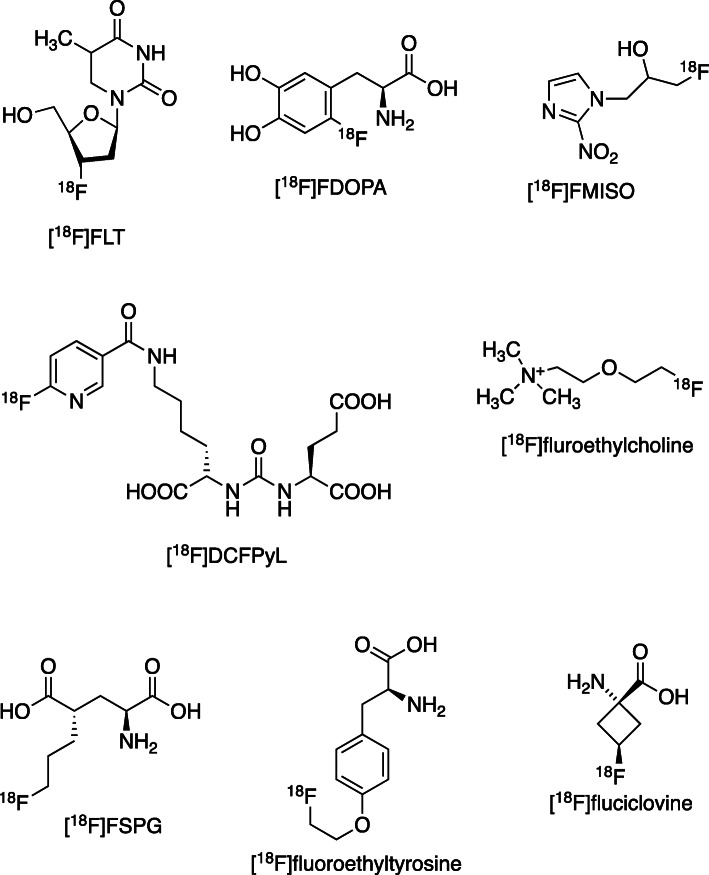
Examples of ^18^F-labelled small molecular imaging agents

The small size of the fluorine atom means that it can be isosterically incorporated into small biologically important molecules by replacing hydrogen atoms or hydroxyl groups (Fig. 3), or indeed stable fluorine atoms already present in numerous drugs. In addition, its covalent bond with carbon generally has high in vivo kinetic stability. These chemical features, alongside its growing availability, make ^18^F an important and attractive radiolabel for an enormous range of potential small molecular radiopharmaceuticals, such as FLT ([^18^F]-3′-fluoro-3′-deoxythymidine, a nucleoside analogue and cell proliferation marker) [21]; F-DOPA ([^18^F]dihydroxyphenylalanine, a dopamine precursor analogue used in neuroendocrine and brain tumours) [22]; F-MISO ([^18^F]fluoromisonidazole, a nitroimidazole hypoxia imaging agent) and related nitroimidazole derivatives [23]; [^18^F]DCFPyL (a prostate-specific membrane antigen (PSMA) ligand used for prostate cancer imaging) and other ^18^F-labelled PSMA ligands [24]; [^18^F]fluoroethylcholine [25] and related tracers for prostate and other tumours; and amino acid analogues such as FET ([^18^F]fluoroethyltyrosine), [26] FSPG (3-[^18^F]fluoropropyl)-L-glutamic acid (glutamate analogue taken up by cells through the system x_C−_ transporter) [27] and [^18^F]fluciclovine [28]; to name but a few (Fig. 4). When these agents are small organic drug- or metabolite-like molecules (as in the case of FDG) there is almost no alternative to fluorine-18 except for carbon-11, the short half-life of which precludes widespread distribution and utility. Accordingly, much effort has been expended in recent decades in developing chemistry – much of it beyond the scope of this review - for incorporation fluorine-18 into small molecules [29].

However, to realise the full potential of PET in modern targeted imaging and immunotherapy, not just small molecules but also peptides and proteins such as antibodies and engineered antibody analogues have a role as targeting agents. Fluorine-18 is less well-suited chemically to addressing this challenge; nevertheless, innovative solutions are currently being sought, as discussed below.

### Peptide and small protein labelling with radiometals – ^99m^Tc, ^68^Ga and “the need for speed”

One solution to the challenge of labelling peptides and proteins under mild conditions is to use a radiometal rather than fluorine-18. Radiometals can, at least in principle, usually be incorporated into biomolecules in a single step, which greatly simplifies labelling.

The first methodology for radiolabelling peptides and proteins with radiometals began to emerge in the 1970s and 1980s, with technetium-99 m [30, 31] and indium-111 before clinical PET became established. Early developments with ^111^In labelling using the prototypical bifunctional chelator DTPA (diethylene triamine pentaacetic acid), [32] initially incorporated into antibodies by reaction of its cyclic anhydride with lysine side chain amino groups, were remarkably successful and were a basis for modifications to improve kinetic stability, allowing extension to more labile radiometals such as yttrium-90, by adding rigidity to the backbone or developing macrocyclic derivatives [33]. The radiolabelling process with these early ^111^In was very simple and efficient, often with sufficient radiochemical yield to avoid the need for purification steps. Indeed, DTPA chelation of ^111^In became the basis of the first commercial peptide radiopharmaceutical, Octreoscan, for imaging somatostatin-receptor expressing tumours [34].

**Fig. 5:**
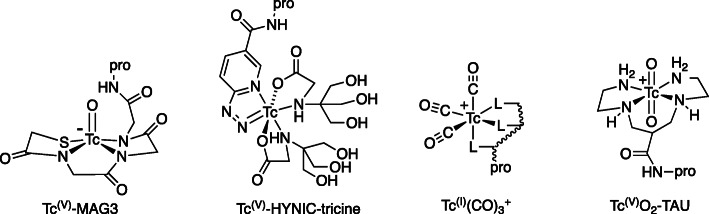
Bifunctional chelators and cores for ^99m^Tc labelling of biomolecules (pro = protein or other biomolecule)

The chemistry of incorporating technetium-99 m into proteins is more complex and entails redox; numerous bifunctional chelators and cores in different oxidations states (Fig. 5) were developed in the 1990s, most prominently tetradentate sulfur/nitrogen ligands for chelation of Tc = O^3+^, [35] HYNIC (hydrazinonicotinamide) derivatives, [36] and Tc(CO)_3_^+^ [37] for attachment via bifunctional chelators or histidine-tags. However, few were amenable to simple one-step labelling or free of other problems such as radiochemical inhomogeneity or in vivo instability, and ^99m^Tc-labelled protein and peptide-based radiopharmaceuticals made little clinical impact for this reason. Before a simpler and more user-friendly ^99m^Tc-radiolabelling chemistry could be developed, the age of molecular imaging with PET had arrived, and technetium chemistry research waned. Since the turn of the millennium, ^99m^Tc chemistry for biomolecule labelling – indeed, for novel radiopharmaceuticals in general - has been almost entirely neglected, as radiochemists turned instead to ^18^F (see above) and ^68^Ga labelling of biomolecules, publications on which have risen dramatically since 2000 as those on ^99m^Tc declined. The most promising peptide-linked bifunctional chelator system to emerge since the millennium is the 1,4,8,11-tetrazaundacane-based chelator (TAU, Fig. 5) [38] which coordinates the TcO_2_^+^ core under mild conditions. It has not yet been widely used.

**Fig. 6:**
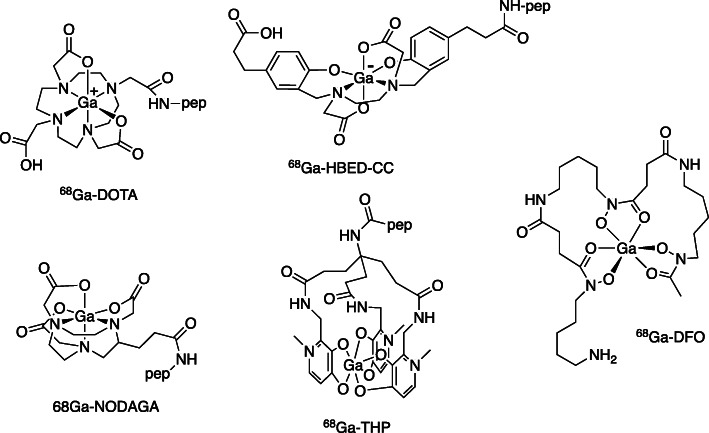
Examples of cyclic and acyclic bifunctional chelators for gallium-68; some are also useful for other radiometals: ^68^Ga-DOTA, ^68^Ga-HBED-CC (one of several isomeric structures), ^68^Ga-NODAGA, ^68^Ga-THP and ^68^GA-DFO; pep = peptide, protein or other biomolecule

Gallium-68 in particular attracted attention for peptide labelling because of the growing availability of the ^68^Ge/^68^Ga generator and the close match of its short (68 min) half-life with the typical blood clearance and excretion kinetics of small peptides and peptide-like molecules. It is unsuitable for labelling whole IgG antibodies because these take much longer to clear, but smaller antibody fragments and engineered analogues clear quickly and are compatible with ^68^Ga [39]. Early bioconjugates of ^68^Ga made use of the 1,4,7,10-tetraazacyclododecane-1,4,7,10-tetraacetic acid (DOTA) bifunctional chelator [40] which, because of the paramount importance of in vivo stability in therapeutic applications of longer half-life radionuclides, had become established through its use with ^111^In and the therapeutic radionuclides yttrium-90, copper-64/67, and later lutetium-177. Consequently, several receptor-targeted ^68^Ga-DOTA derivatives have gradually come into clinical use [41]. However, the slow pace of clinical adoption of such ^68^Ga tracers has revealed an unfortunate consequence of the way in which PET tracers have developed since the arrival of clinical PET: the inevitable complexity of the multi-step synthesis of ^18^F-labelled small molecules has led to acceptance that PET radiolabelling generally is complex and requires costly infrastructure, limiting access to these tracers for patients and imposing prolonged (typically months) and demanding validation at each user site. ^68^Ga labelling of DOTA-conjugates requires high temperature (90 °C), long reaction times (30 min) and low pH, and often gives low molar activity or yield that necessitates a purification step – all of which add complexity that has become accepted. The experience of “conventional” radiopharmacy, making extensive use of ^99m^Tc in very simple kit-based labelling procedures, has taught us that this need not be so: with more efficient labelling chemistry, it should in principle be possible to perform fast, efficient, kit-based labelling with ^68^Ga with minimal equipment and operator time. The limiting factor is the design of the chelating agent. This realisation spawned numerous designs for bifunctional chelators [41] intended to improve on the labelling kinetics and conditions of DOTA. Thus emerged new bifunctional chelators that can be labelled quickly at room temperature without extremes of pH or need for purification, and hence are amenable to kit-based labelling, offering wider availability and patient benefit. The most effective of these, which include both cyclic (NOTA - 1,4,7-triazacyclononane-N,N′,N″-triacetic acid - derivatives and their phosphate analogues) [42] and acyclic (HBED - N,N′-Di(2-hydroxybenzyl)ethylenediamine-N,N′-diacetic acid, [43] THP – tris(hydroxypyridinone) [44]) chelators, have found wide clinical use (e.g. in tracers targeting somatostatin receptors in neuroendocrine tumours, and PSMA in prostate cancer [45]); examples are shown in Fig. 6. In the case of THP, the labelling conditions are sufficiently mild that simple kit-based labelling of small proteins and antibody fragments is feasible in minutes [39].

The short half-life of gallium-68 means that production and radiolabelling at or near the site of use is necessary, and the ^68^Ge/^68^Ga generator makes this possible. In economic or geographic contexts where a longer half-life equivalent is needed, allowing transport of the radiopharmaceutical between sites, scandium-44 (half-life 4.0 h) is emerging as a potential cyclotron- or generator-produced alternative [46]. It can, potentially, be produced in many hospital cyclotrons and readily attached to DOTA-peptide conjugates. If a clinical role for scandium-44 becomes established for these reasons, there may be a need for optimised scandium-specific chelators to be developed.

### Chelators and fluorine – inorganic fluorine chemistry

**Fig. 7:**
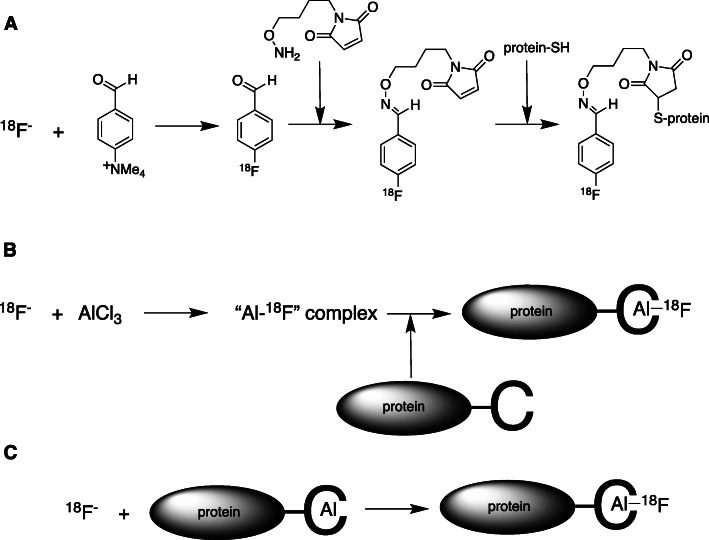
^18^F-labelling of biomolecules – the problem and a putative solution. **a**: Conventional organic prosthetic group labelling is a multistep sequence of reactions; **b**: Aluminium-fluoride coordination approach: still multi-step; **c**: idealised one-step approach: not yet achieved under mild conditions

Fluorine-18 is an attractive choice for labelling peptide and small protein-based tracers because of the good match of its half-life with the typical time-scale of scanning combined with its widespread economic availability. However, from a chemical perspective, labelling these biomolecules using [^18^F]fluoride is extremely challenging [47]. Efficient formation of a stable C-F bond as the final step in synthesis in a biomolecule that cannot tolerate extreme chemical conditions and contains many functional groups that are more nucleophilic than fluoride ions, is currently impossible. The conventional solution necessitates multi-step radiolabelling, whereby an organic prosthetic group, such as fluorobenzoic acid, fluorobenzaldehyde or a click chemistry component, [47] is first radiolabelled, and then incorporated into the biomolecule (Fig. 7a). This complexity, and the accompanying inefficiency, is a significant barrier to wider application of ^18^F-labelled biomolecules.

An innovative range of new chemical approaches to this problem has been opened up by recognising that elements other than carbon can be binding sites for fluoride. Boron, silicon and a range of metal ions have been the focus of attempts to incorporate them into biomolecules to serve as selective high-affinity binding sites for fluoride ions, leading to inventive chemistry but, as yet, no general solution [48]. The most widely adopted approach uses aluminium complexes with NOTA-like pentadentate chelators that leave one ligand binding site on the Al^3+^ ion free to bind a fluoride ion [49]. The kinetics of incorporation of fluoride ions are, however, sub-optimal, and the goal of incorporating [^18^F]fluoride into a pre-formed aluminium-chelate-biomolecule conjugate as the final synthetic step (Fig. 7c) remains out of reach. Instead, the [^18^F]fluoride is first complexed with aluminium, and the resulting complex then added to the biomolecule-chelator conjugate, in a multi-step reaction (Fig. 7b) with little or no advantage over conventional organic ^18^F-labelled prosthetic groups. The search for a metal-chelate fluoride-binding site that can be conjugated to biomolecules and then labelled with [^18^F]fluoride continues, with gallium and iron bound within triazacyclononane-derived ligands currently at the forefront of development [50].

### Theranostics – the example of copper

Early interest in incorporation of copper radionuclides into radiopharmaceuticals [51] focussed on use of copper-67, a beta-emitter with a half-life of 61.8 h, in radioimmunotherapy. The long half-life of ^67^Cu matches the clearance kinetics of monoclonal antibodies and long-term in vivo stability is the most pressing demand in the design of bifunctional chelators. Despite great therapeutic promise, interest in this application faded because of unreliable and costly production methods for the radionuclide, and is only very recently recovering as more economically feasible production methods emerge.

**Fig. 8:**
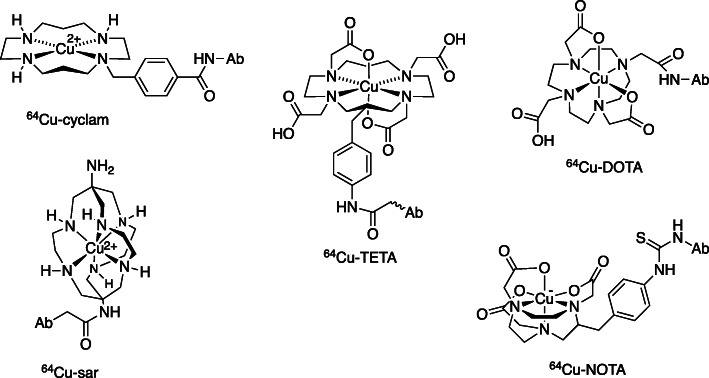
Bifunction chelators for ^64/67^Cu^2+^. Each is drawn as an exemplar conjugated to an antibody (Ab) or other biomolecule

As clinical PET developed, interest turned to the copper-64, which can be produced using a typical biomedical cyclotron. ^64^Cu is both a beta- and positron- (and Auger electron-) emitter lending itself to both PET and therapeutic application. A “theranostic” pairing, in which ^64^Cu PET imaging provides quantitative and accurate biodistribution data to underpin dosimetry for therapeutic use of ^64^Cu and ^67^Cu, can be envisaged. The half-life of ^64^Cu is 12.7 h, reasonably compatible with antibodies, peptides and small molecules and sufficiently long that rapid labelling is less important than in vivo stability. Because Cu^2+^ is intrinsically more kinetically labile than other radiometals discussed above, no acyclic chelators have found use for ^67^Cu and ^64^Cu. Against this background, macrocyclic bifunctional copper chelators (Fig. 8) have developed from cyclam and TETA (1,4,8,11-tetraazacyclotetradecane-N,N′,N″,N″‘-tetraacetic acid) in the early 1990s, through DOTA and cross-bridged analogues of TETA designed to enhance kinetic stability, [51] recently settling on NOTA and the cage-like sarcophagine (sar) derivatives (Fig. 8) as the preferred chelators, [52] with a suitable balance of facile labelling and excellent long-term in vivo stability for use with ^64^Cu and ^67^Cu, for labelling peptides, antibodies and small proteins. On the other hand, for the generator-produced copper-62 (half-life 9 min) the priorities are reversed: fast labelling is paramount while prolonged in vivo stability is of little consequence.

### ImmunoPET – zirconium-89 and manganese-52

A core demand of imaging in the age of targeted and immune therapy is tracking the fate of antibodies, whether used therapeutically, diagnostically or theranostically. Radiolabelling antibodies has been a focus of radiochemists since the 1980s, as highlighted above, but the demand to extend imaging with antibodies from gamma scintigraphy to PET has highlighted the need for accessible long half-life positron-emitting radionuclides. Zirconium-89 (half-life 3.3 days) [53] and iodine-124 (half-life 4.2 days) [54] have emerged to meet this need. Stability of the antibody-radiolabel link is paramount; speed of labelling, less so. In the case of ^89^Zr a suitable bifunctional chelator emerged quickly and fortuitously without the long years of trial and optimisation of dozens of ligand systems that characterised the development of copper bifunctional chelators. Desferrioxamine-B (DFO, Fig. 6), a chelator designed by nature (in the evolution of bacteria and fungi) for iron(III) chelation, has shown adequate in vivo stability while being amenable to simple, efficient radiolabelling under mild conditions [55]. With a naturally-present pendant primary amine, it is easily converted to reactive forms that can be conjugated to proteins via primary amine and thiol groups of amino acid side chains. Because DFO is hexadentate, while the Zr^4+^ ion is quite large and typically found in eight-coordinate structures, several groups have supposed that DFO is sub-optimal as a ^89^Zr-chelator and have sought extended octadentate versions of DFO to fully encapsulate the metal and enhance resistance to transchelation in vivo. However, it is hard to find clear evidence that transchelation or release of ^89^Zr occurs to a significant extent in vivo. Free zirconium accumulates in bone, and the now very numerous immunoPET scans performed in humans with antibody-DFO conjugates labelled with ^89^Zr have not shown off-target uptake in bone [55]. Despite the additional practical challenges associated with longer half-life radionuclides, including higher patient radiation doses and additional demands on handling, disposal and avoidance of contamination, immunoPET with ^89^Zr-DFO-conjugates is a success story of recent years. It has led to excellent commercial availability of zirconium-89 and perhaps rendered many years of work on ^64^Cu antibody labelling obsolete – this remains to be seen – since it is cheaper to produce and its long half-life is a better match to the required scanning times and makes distribution from supplier to user easier. Iodine-124, although in principle amenable to protein-labelling chemistry that is well-established (having been developed many years ago for application to gamma emitters ^131^I, ^125^I and ^123^I), has not enjoyed such widespread use so far.

Should it prove necessary or useful to extend the immunoPET scanning times for longer still, a newly emerging longer-lived radionuclide with similar emission properties, manganese-52 (half-life 5.6 days), may provide the solution. The first ^52^Mn-immunoconugates, labelled without undue difficulty by means of the DOTA (Fig. 6) bifunctional chelator, [56] have shown the requisite in vivo stability. It remains to be seen whether DOTA can, or needs to be, improved upon for this purpose.

### Cell tracking

Among the armoury of emerging immunotherapeutics are cell-based therapies such as mesenchymal stem cells and tumour-targeted CAR-T cells. The risks of adverse effects of these therapies, and the need to understand reasons for their success or failure, have highlighted the need for clinical trials to incorporate methodology to track the migration, survival and proliferation of administered therapeutic cells [5] and to enumerate cells reaching the target or off-target tissues.

Tracers to enable the reporter gene approach to cell tracking were briefly introduced earlier in this review; this approach has recently been used in humans to track cytotoxic T lymphocytes carrying a non-human reporter gene, the herpes simplex virus type 1 thymidine kinase (HSV1-TK), by PET with nucleoside analogue 9-[4-[^18^F]fluoro-3-(hydroxymethyl)butyl]guanine ([^18^F]FHBG [57].

**Fig. 9:**
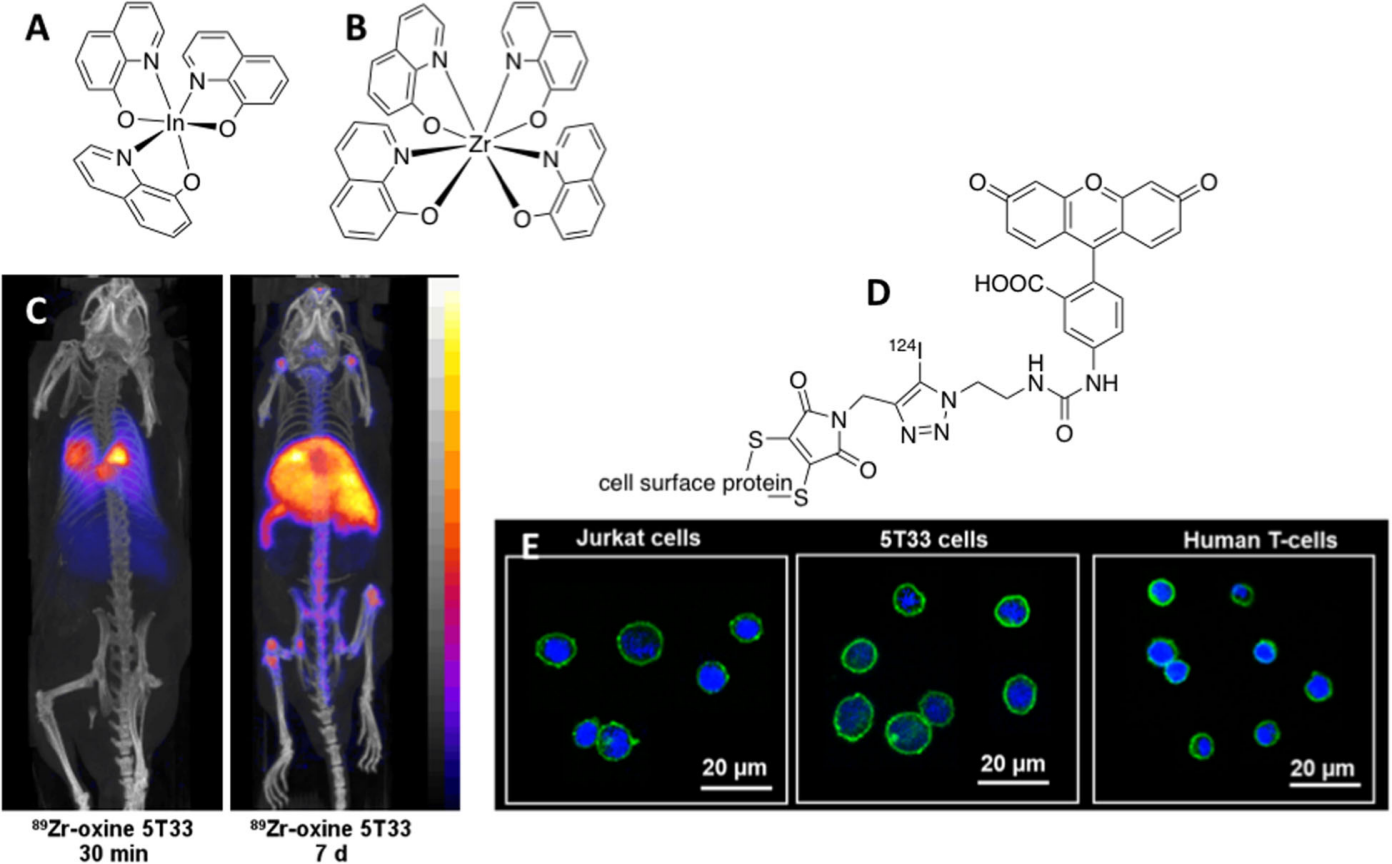
Cell tracking by radionuclide imaging. **a**: ^111^In-oxine complex for intracellular labelling of cells for SPECT imaging; **b**: ^89^Zr-oxine complex for intracellular labelling cells for PET imaging; **c**: PET imaging of 5 T33 melanoma cells in mouse using the ^89^Zr reagent shown in B, 30 min and 7 days after i.v. injection of cells; **d**: ^124^I-labelled fluorescent tracer for cell-surface protein labelling cells for PET imaging; **e**: Fluorescence microscopy of Jurkat, 5 T33 melanoma and human T lymphocytes labelled with ^124^I reagent shown in D, showing cell surface localisation (green) of the tracer

The simplest approach, complementary to reporter gene imaging, is to administer radiolabelled cells, as has been done routinely in clinical nuclear medicine since the 1980s to localise inflammation and infection using white blood cells labelled with indium-111 [58]. However, the advantages of PET over scintigraphy have prompted development of long half-life positron-emitting cell labelling agents. First and most promising among these is zirconium-89, incorporated into cells by means of a metastable lipophilic complex of 8-hydroxyquinoline or “oxine”, [Zr(oxinate)_4_] (Fig. 9) [59]. This labelling reagent and method is analogous to the indium-111-oxine complex [In(oxinate)_3_] and works by a similar mechanism, involving intracellular transchelation, and hence trapping, of zirconium. This allows imaging of cell migration for as much as 1–2 weeks, since the radionuclide is largely retained by the labelled cells. A simplified, good manufacturing practice (GMP)-compatible kit-based method of producing the ^89^Zr-oxine complex has been reported recently, [60] greatly enhancing the opportunity for wider use in clinical trials of cell-based therapy. An alternative approach to cell labelling is by conjugation of DFO-based zirconium-89 chelates [61] or iodine-124-prosthetic groups [62] (Fig. 9) to proteins on the cell surface. [^89^Zr(oxinate)_4_], and analogous [^64^Cu(oxinate)_2_] and [^52^Mn(oxinate)_2_], have also been used to label liposomal drug formulations for PET imaging of their biodistribution [63].

### New challenges and unmet needs

The above discussion has highlighted examples in which radiochemistry challenges posed by new needs for molecular imaging to support advances in targeted and immunotherapies have been met by means of radionuclide imaging. Methods have been developed for production of effective radiolabelled small molecule-, peptide-, protein-, nanoparticle- and cell-based probes for a wide array of targets. In some areas there are obstacles still to be overcome. Despite the rise of PET, and the shortages of ^99^Mo/^99m^Tc generators besetting the nuclear medicine community in the last decade or so, the great majority of daily radionuclide imaging around the world is still conducted using technetium-99 m scintigraphy and SPECT. The “kit” approach to radiopharmaceutical production originated decades ago with technetium-99 m, and has been extended in recent years to make gallium-68 labelling of sensitive biomolecules simple and quick; yet we have no established easy-to-use (that is, single-step, kit based) technetium-99 m radiochemistry for labelling proteins and peptides; methods developed in the 1990s fall short of this ideal and development since 2000 has been negligible. This decline must be reversed if advances in molecular imaging are to benefit the maximum number of patients worldwide.

Similarly, the wide availability of fluorine-18 has made possible clinical use of a variety of small organic molecular tracers for PET, but the complexity of their synthesis and the associated need for high-capital value facilities and expert radiochemistry staff has limited widespread use of all but a few, and made them very cumbersome to use with proteins. Consequently ^18^F-labelled proteins have made little impact in the clinic and ^18^F-labelled peptides are only marginally more established. One-step labelling of these biomolecules using [^18^F]fluoride under mild, aqueous conditions remains sought-after but elusive.

The many radiochemical innovations described above have been driven not only by the wide range of molecular targets associated with targeted therapy and immunotherapy in cancer, but also by developments in imaging physics which change the expectations of what can be achieved. Most obviously, the availability of PET-CT (PET-computed tomography) scanners and cyclotrons, in turn driven by the clinical importance of FDG, has catalysed production of new radionuclides and development of new radiochemistry to exploit the advantages of PET over scintigraphy and SPECT. The advent of PET-MR (PET-magnetic resonance) has also contributed to new combined modality tracer design [64].

Despite the plethora of molecular targets of interest to imagers to help characterise disease heterogeneity and treatment response, radionuclide imaging of more than one of the molecular characteristics in an individual patient, as outlined in the introduction, is fraught with difficulties including exceeding acceptable radiation doses, the inconvenience of multiple visits for scanning, and extended use of scanner time. Now a new technology is emerging which promises to be a game-changer in this respect, perhaps as important as the advent of clinical PET itself – Total Body PET [65]. The ability to image the whole body simultaneously, and the associated order-of-magnitude increase in sensitivity, reduction in scan time and improvement in resolution, allows not only reduced radiation doses and higher patient throughput, but also the possibility to image multiple phenotypes using several tracers in one patient, allowing a much deeper molecular characterisation of disease across the whole body. This too has the potential to be a new driver for innovative radiochemistry. Cell tracking with PET will become more attractive and feasible using lower quantities of the long half-life radionuclides such as zirconium-89, with greater confidence in the results because of the reduced radiation absorbed dose to the labelled cells, or more prolonged tracking (weeks) of the cells when using comparable doses to conventional PET. The possibility of performing multiple scans with different tracers will stimulate development of a wider range of radiopharmaceuticals, with a greater emphasis on short half-life radionuclides, so that scans can be performed sequentially in tandem. One can envisage sequential use of tracers such as rubidium-82 (characterising blood flow and sodium-potassium ATPase activity in tumours), peptides and hypoxia tracers labelled with generator-produced copper-62 (half-life 9 min), [51] small molecules labelled with nitrogen-13 (half-life 10 min) [29] and carbon-11 (half-life 20 min), [29] combined with conventional radiopharmaceuticals such as FDG and labelled antibodies and cells. This brings into even sharper focus the need for speed and simplicity in radiolabelling methodology.

## Conclusion

In the interdisciplinary world of nuclear medicine and molecular imaging, as quickly as radiochemistry solutions are developed to meet new needs in molecular imaging, new challenges emerge, driven both by the advances in therapy they support and by parallel advances in scanning technology, as developments in one contributing technology drive innovations in the others.

## Data Availability

Not applicable.

## Abbreviations

PET: Positron emission tomography
SPECT: Single photon emission computed tomography
NIS: Sodium-iodide symporter
hNIS: Human sodium-iodide symporter
CAR: Chimeric antigen receptor
FDG: [^18^F]-2-Fluoro-2-deoxyglucose
GLUT: Glucose transporter
FLT: [^18^F]-3′-Fluoro-3′-deoxythymidine
F-DOPA: [^18^F]Dihydroxyphenylalanine
F-MISO: [^18^F]Fluoromisonidazole
DCFPyL: 2-(3-{1-Carboxy-5-[(6-[(18)F]fluoro-pyridine-3-carbonyl)-amino]-pentyl}-ureido)-pentanedioic acid
FET: [^18^F]Fluoroethyltyrosine
DTPA: Diethylene triamine pentaacetic acid
HYNIC: Hydrazinonicotinamide
TAU: 1,4,8,11-Tetrazaundacane
DOTA: 1,4,7,10-Tetraazacyclododecane-1,4,7,10-tetraacetic acid
NOTA: 1,4,7-Triazacyclononane-N,N′,N″-triacetic acid
THP: Tris(hydroxypyridinone)
sar: Sarcophagine
DFO: Desferrioxamine-B
HSV1-TK: Herpes simplex virus type 1 thymidine kinase
FHBG: 9-[4-Fluoro-3-(hydroxymethyl)butyl]guanine
GMP: Good manufacturing practice
CT: Computed tomography
MR: Magnetic resonance
ATP: Adenosine triphosphate
